# The efficacy and safety of PARP inhibitors in mCRPC with HRR mutation in second-line treatment: a systematic review and bayesian network meta-analysis

**DOI:** 10.1186/s12885-024-12388-2

**Published:** 2024-06-08

**Authors:** Qiyu Zhu, Junru Chen, Haoyang Liu, Jinge Zhao, Chenhao Xu, Guangxi Sun, Hao Zeng

**Affiliations:** Department of Urology, Institute of Urology, West China Hospital of Sichuan University, No. 37, Guoxue Alley, Chengdu, Sichuan 610041 P.R. China

**Keywords:** Poly (ADP- ribose) polymerase inhibitors, Homologous recombination deficiency, Metastatic castration-resistance prostate cancer, Progression-free survival, Adverse events

## Abstract

**Background:**

Poly (ADP- ribose) polymerase inhibitors (PARPi) has been increasingly adopted for metastatic castration-resistance prostate cancer (mCRPC) patients with homologous recombination repair deficiency (HRD). However, it is unclear which PARPi is optimal in mCRPC patients with HRD in 2nd -line setting.

**Method:**

We conducted a systematic review of trials regarding PARPi- based therapies on mCRPC in 2nd -line setting and performed a Bayesian network meta-analysis (NMA). Radiographic progression-free survival (rPFS) was assessed as primary outcome. PSA response and adverse events (AEs) were evaluated as secondary outcomes. Subgroup analyses were performed according to specific genetic mutation.

**Results:**

Four RCTs comprised of 1024 patients (763 harbored homologous recombination repair (HRR) mutations) were identified for quantitative analysis. Regarding rPFS, olaparib monotherapy, rucaparib and cediranib plus olaparib showed significant improvement compared with ARAT. Olaparib plus cediranib had the highest surface under cumulative ranking curve (SUCRA) scores (87.5%) for rPFS, followed by rucaparib, olaparib and olaparib plus abiraterone acetate prednisone. For patients with BRCA 1/2 mutations, olaparib associated with the highest probability (98.1%) of improved rPFS. For patients with BRCA-2 mutations, olaparib and olaparib plus cediranib had similar efficacy. However, neither olaparib nor rucaparib showed significant superior effectiveness to androgen receptor-axis-targeted therapy (ARAT) in patients with ATM mutations. For safety, olaparib showed significantly lower ≥ 3 AE rate compared with cediranib plus olaparib (RR: 0.72, 95% CI: 0.51, 0.97), while olaparib plus cediranib was associated with the highest risk of all-grade AE.

**Conclusion:**

PARPi-based therapy showed considerable efficacy for mCRPC patients with HRD in 2nd -line setting. However, patients should be treated accordingly based on their genetic background as well as the efficacy and safety of the selected regimen.

**Trial registration:**

: CRD42023454079.

**Supplementary Information:**

The online version contains supplementary material available at 10.1186/s12885-024-12388-2.

## Introduction

Prostate cancer (PCa) is the most commonly diagnosed cancer in men, accounting for over 10% of all cancer-caused death in 2023 [1]. Metastatic castration-resistant prostate cancer (mCRPC) was considered as the terminal stage of PCa with a median overall survival of less than 3 years [2–4]. Androgen receptor-axis-targeted (ARAT) regimens and taxane have been widely used as the first-line treatments for mCRPC in recent years [5, 6]. However, patients inevitably experience progression after receiving these agents [7–9], thus the exploration of effective 2nd -line therapy for mCRPC experiencing treatments failure becomes increasingly important.

With the rapid development of precision medicine, molecular characterization of PCa has led to the discovery of multiple actionable genomic alterations. Several large-scale studies have revealed that 20-30% of mCRPC patients harbored germline or somatic DNA damage repair (DDR) mutation, including those participating in homologous recombination repair (HRR) pathway, which is targeted by poly (ADP- ribose) polymerase inhibitors (PARPi) [10, 11]. PARPi functions through selectively binding catalytic pocket among PARP1/2 and DNA trapping to achieve DNA damage repair inhibition, and thus induce synthetic lethality specifically in patients with homologous recombination deficiency (HRD) [12–14].

Multiple trials exploring the efficacy of PARPi in 2nd -line mCRPC setting have been carried out and demonstrated promising anti-tumor activity especially in HRD patients [14–18]. Several previous meta-analyses and systematic reviews also confirmed the superior efficacy of PARPi-based therapies in mCRPC patients with HRD [19–21]. However, no head-to-head comparative trials of different PARPis have been conducted, and the optimal PARPi-based treatment for this population remains unknown. Thus, we conducted this systematic review and network meta-analysis (NMA) to assess the efficacy and safety of different PARPis in 2nd -line mCRPC setting with HRD.

## Method

This network meta-analysis adhered to the guidelines of PRISMA for Network Meta-Analyses (PRISMA-NMA) and adopted the standard methods approved by the Cochrane Collaboration [22, 23]. The protocol of this NMA was registered on PROSPERO in prior (CRD42023454079).

### Literature research

A systematic literature review was conducted based on three databases (PubMed, Cochrane CENTRAL, and Embase), focusing on papers published before August 2, 2023. Detailed search strategy was attached to the protocol published on PROSPERO. Two reviewers (HYL and QYZ) were responsible for the literature scanning process based on title, abstract, and full text. Disagreements were resolved under the guidance of a third reviewer (JRC).

### Eligibility criteria

Studies meeting the following eligibility criteria were included in this NMA: (1) Trials comparing PARPi with androgen receptor axis-targeted (ARAT) agents or combination therapy of PARPi and other anti-tumor regimens (e.g., abiraterone, cediranib). (2) mCRPC patients progressed after first-line treatment. (3) Randomized controlled trials. (4) Studies reporting radiographic progression-free survival (rPFS). Research of references of included studies was carried out and cross-referenced manually for potentially eligible articles. Case reports, single-arm trials, meeting abstracts and reviews were excluded. No language restrictions were applied. Detailed searching strategies are displayed in Additional file 1.

### Data extraction and quality assessment

Two independent reviewers (HYL and QYZ) were responsible for data extraction using the standardized format developed in prior, including the year of publication, author, registry of the included RCTs, study phase, mutational status of the cohort, number of patients, performance status, treatment level, previous treatment, age, baseline PSA level and outcome measures. Assessment of risk of bias was conducted using the Cochrane risk-of-bias (RoB2) tool [24]. Studies were assessed in the following five domains: (1) Randomization process. (2) Deviations from intended intervention. (3) Missing outcome data. (4) Measurement of the outcome. (5) Selection of the reported result. The overall risk of bias of a study was accessed as ‘low risk of bias’ when all domains were judged as ‘low risk of bias’, ‘some concern’ when at least one domain was judged as ‘some concern’ and ‘high risk of bias’ when at least one domain was judged as ‘high risk of bias’. Discrepancy in data extraction and quality assessment was resolved through discussion.

### Study outcomes

The primary outcome was rPFS, which was defined as the time from randomization to disease progression or death. Secondary outcomes included PSA response, defined as ≥ 50% decline in the concentration of PSA level, and adverse events (AEs). Subgroup analyses were conducted according to specific HRR mutations.

### Statistical analysis

For rPFS, we performed Bayesian NMA using log hazard ratio (HR) and standard error calculated based on HR and 95% CI supplied in the original article. For PSA response and AEs, the number of events of each treatment group was collected for estimation of risk ratio (RR) and the corresponding 95%CI. Random-effects or fixed-effects model was adopted based on heterogeneity assessed by I^2^ with four parallel Markov chains consisting of 50,000 iteration phase and Bayesian deviance information criterion (DIC). I^2^ > 50% was considered to indicate significant heterogeneity. Convergence was assessed using trace plots and Gelman-Rubin-Brooks plots. We also performed a calculation of surface under the cumulative ranking (SUCRA) to rank preferences of different regimens in each outcome. Sensitivity analysis was conducted using a random-effects model by calculating SUCRA values for each outcome. All statistical analyses were performed using R (version 4.3.1).

## Result

### Included studies and characteristics

**Fig. 1:**
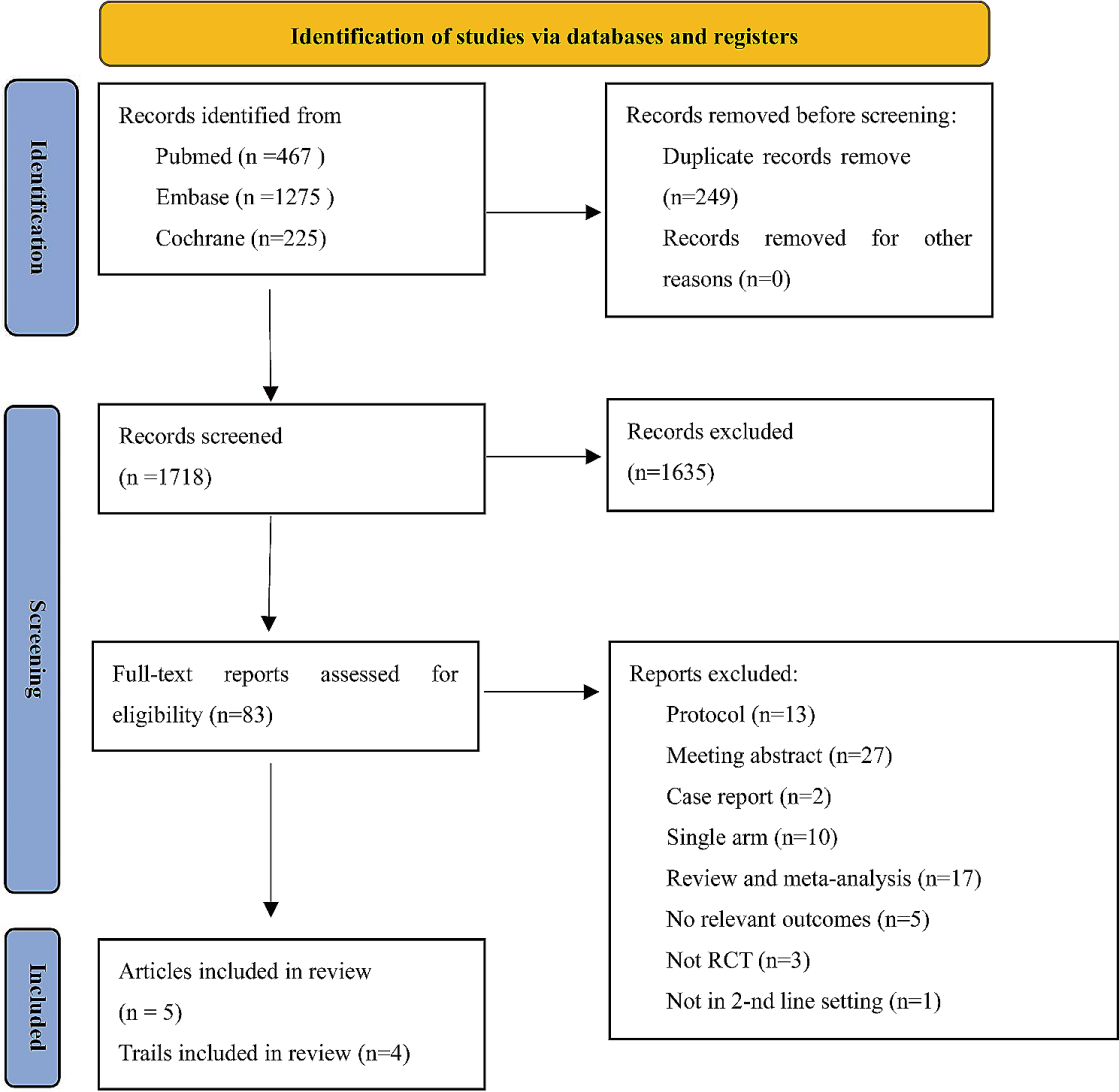
Literature search and study selection process according to PRISMA flow diagram for systematic reviews

**Fig. 2:**
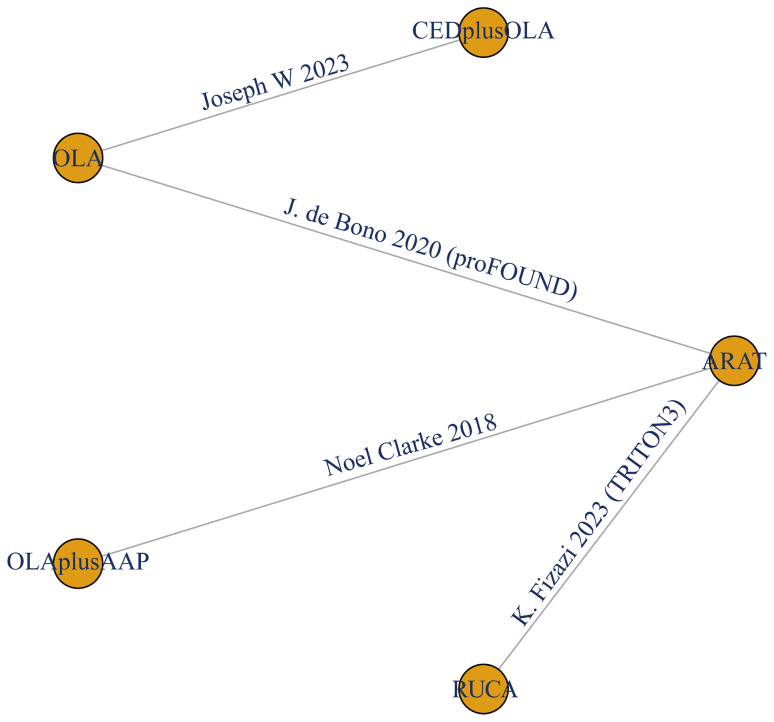
Network plot of the efficacy comparisons among different poly (ADP- ribose) polymerase inhibitors and androgen receptor-axis-targeted therapy (ARAT) regarding radiographic progression-free survival. Abbreviation: CED: cediranib; OLA: olaparib; AAP: abiraterone acetate plus prednisone; RUCA: rucaparib

A total of 1967 studies were retrieved after comprehensive literature research and 1718 studies were identified after duplicates removal. After title and abstract screening, we conducted a full-text review of 83 articles. Finally, 4 RCTs with 5 articles comprised of 1024 patients (763 with HRR mutations) were eligible for this network meta-analysis **(**Fig. 1**)**. Among the included trials, two RCTs used PARPi alone (olaparib, rucaparib) [9, 25] and one RCT adopted olaparib in combination with abiraterone acetate plus prednisone (AAP) as the intervention [17]. ARAT/ARAT + placebo was selected as control treatment in the above three studies. One trial, which contained patients receiving more than one regimen prior to recruitment (≥ 2 line), assessed the effect of cediranib plus olaparib compared to olaparib monotherapy **(**Fig. 2**)** [16]. None of the included studies reported significant between-group differences in baseline patient characteristics. Detailed characteristics of the included trials are summarized in Table 1.

**Table 1:** Baseline characteristics of the four randomized controlled trials for Bayesian network meta-analysis

Study	Registry	Treatment	Study phase	Cohort requirement	Patients in total cohort	Patients in HRRm cohort	Performance status	Treatment level	Previous drug adoption	Age (yr), median (range)	PSA (ng/ml), median (range)	Median OS (months)	Median rPFS (months)
Johann de Bono 2020 (proFOUND)	NCT02987543	Olaparib	III	HRR deficiency	256	256	ECOG ≤ 2	2nd-line	Second-generation ARAT/taxane	69 (47–91)	68.2 (24.1–294.4) ^ø^	17.3	5.8
		ARAT (enzalutamide or abiraterone)			131	131				69 (49–87)	106.5 (37.2–326.6) ^ø^	14	3.5
Karim Fizazi 2023 (TRITON3)	NCT02975934	Rucaparib	III	HRR deficiency (BRCA/ATM)	270	270	ECOG ≤ 2	2nd-line	Second-generation ARAT	70 (45–90)	26.9 (0.1–1247)	23.6	10.2
		Docetaxel or a second-generation ARAT [abiraterone acetate or enzalutamide]			135	59				71 (47–92)	28.8 (0–1039)	20.9 ^þ^	4.5
Joseph W. Kim 2023	NCT02893917	Cediranib + olaparib	II	No requirement	45	12	ECOG ≤ 1	≥ 2nd-line	Second-generation ARAT/taxane/sipuleucel T	66 (48–81) ^*^	62 (0.30-3,145) ^*^	11.77 ^*^	10.63
		Olaparib			45	14				70 (51–82) ^*^	51 (0.02-1,453) ^*^	17.37 ^*^	3.83
Noel Clarke 2018	NCT01972217	Olaparib + AAP	II	No requirement	71	11	ECOG ≤ 2	2nd-line	Taxane	70 (65–75) ^*^	86 (23–194) ^* ø^	22.7 ^*^	17.8
		ARAT (placebo + AAP)			71	10				67 (62–74) ^*^	47 (21–199) ^* ø^	20.9 ^*^	6.5

* The data were deprived of total cohort due to lack of corresponding estimation for HRRm cohort; ø data were measured in median (IQR); þ the data were estimated in cohort in which participants were treated with ARAT or docetaxel. Abbreviation: HRRm: homologous recombination repair mutated; ARAT: androgen receptor-axis-targeted therapy; yr: year; PSA: prostate specific antigen; OS: overall survival; rPFS: radiographic progression-free survival; HRR: homologous recombination repair

### Network meta-analysis

#### rPFS

**Fig. 3:**
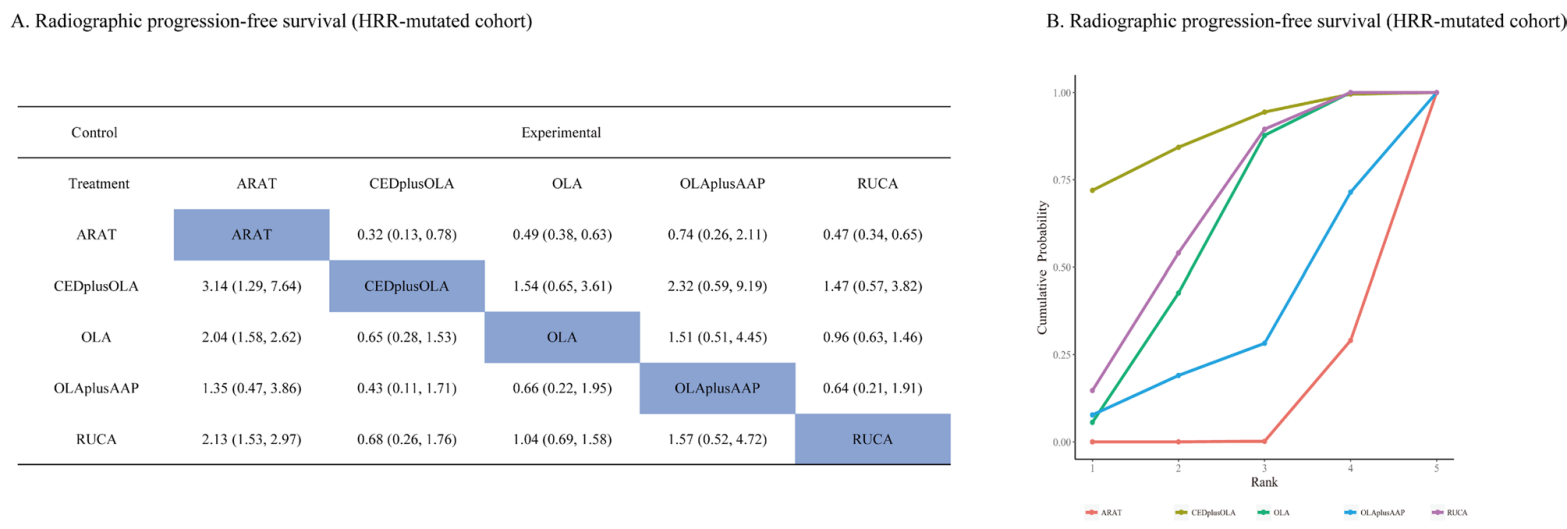
Pairwise comparison and SUCRA for radiographic progression-free survival in HRR-mutated population based on Bayesian network meta-analysis. (**A**): Pairwise comparison for radiographic progression-free survival in HRR-mutated population; (**B**): SUCRA for radiographic progression-free survival in HRR-mutated population. Abbreviation: SUCRA: surface under cumulative ranking; HRR: homologous recombination repair; ARAT: androgen receptor-axis-targeted therapy; CED: cediranib; OLA: olaparib; AAP: abiraterone acetate plus prednisone; RUCA: rucaparib

A total of four studies reported rPFS for mCRPC patients with HRD. Cediranib plus Olaparib showed a significant improvement in rPFS (hazard ratio (HR): 0.32, 95% CI: 0.13, 0.78) compared to ARAT, as did the olaparib monotherapy (HR: 0.49, 95% CI: 0.38–0.63) and rucaparib (HR: 0.47, 95% CI: 0.34–0.65). Olaparib plus AAP (HR: 0.74, 95%CI: 0.26, 2.11) showed no improvement in rPFS **(**Fig. 3A**)** over ARAT alone. The SUCRA suggested cediranib plus olapairib hold a probability of 87.5% to be the most effective regimen, followed by rucaparib (64.6%), olaparib (59.0%) and olaparib plus AAP (31.6%) **(**Fig. 3B**)**. Sensitivity analysis showed consistent SUCRA ranking (Additional file 2: Figure S1A).

#### Subgroup analysis: BRCA 1/2, BRCA-2 and ATM

**Fig. 4:**
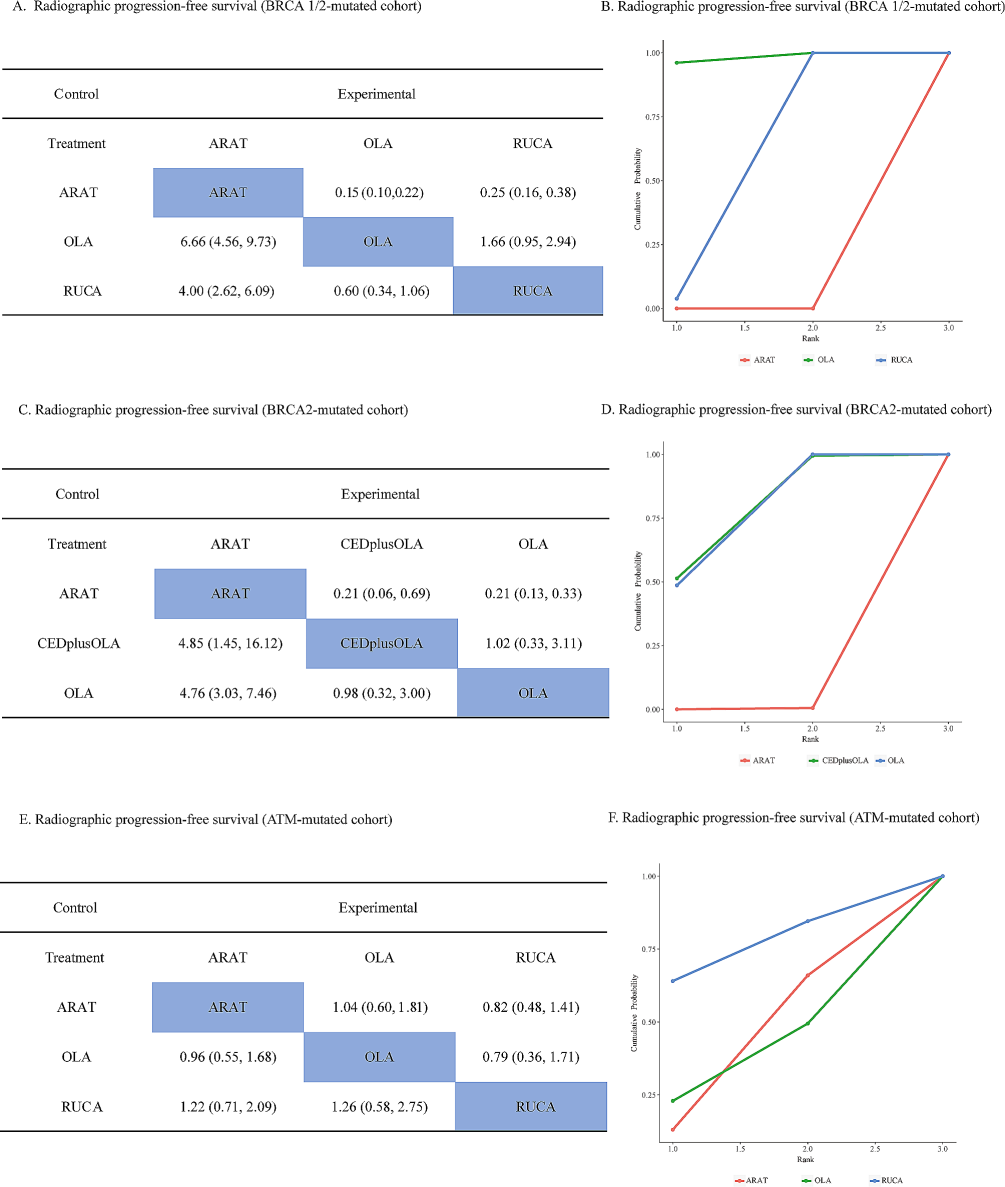
Pairwise comparison and SUCRA for radiographic progression-free survival in subgroup analysis based on genetic mutation. (**A**): Pairwise comparison for radiographic progression-free survival in BRCA 1/2-mutated population; (**B**): SUCRA for radiographic progression-free survival in BRCA 1/2-mutated population; (**C**): Pairwise comparison for radiographic progression-free survival in BRCA-2-mutated population; (**D**): SUCRA for radiographic progression-free survival in BRCA-2-mutated population; (**E**): Pairwise comparison for radiographic progression-free survival in ATM-mutated population; (**F**): SUCRA for radiographic progression-free survival in ATM-mutated population. Abbreviation: SUCRA: surface under cumulative ranking; ARAT: androgen receptor-axis-targeted therapy; CED: cediranib; OLA: olaparib; RUCA: rucaparib

Two studies reported efficacy data of BRCA 1/2-mutated population. Both olaparib (HR: 0.15, 95% CI, 0.10, 0.22) and rucaparib (HR: 0.25, 95% CI, 0.16–0.38) showed significant improvement in rPFS **(**Fig. 4A**)**, with olaparib monotherapy holding higher SUCRA value of 98.1% to be ranked superior over rucaparib (51.9%) **(**Fig. 4B**)**. Two studies reported efficacy data for patients with BRCA-2 mutation. Network analysis showed cediranib plus olaparib (HR: 0.21, 95% CI: 0.06–0.69) and olaparib monotherapy (HR: 0.21, 95% CI: 0.13–0.33) were both associated with improved rPFS **(**Fig. 4C**)** with similar probability of ranking first (cediranib plus olaparib: 75.5%; olaparib: 74.3%, Fig. 4D). Two studies reported survival outcomes in patients with ATM mutation. However, no significant survival improvement was observed for olaparib (HR: 1.04, 95% CI, 0.60–1.81) and rucaparib (HR: 0.82, 95% CI, 0.48–1.41) **(**Fig. 4E**)**. Sensitivity analysis showed similar therapeutic ranking for BRCA 1/2, BRCA-2 and ATM cohort regarding SUCRA values (Additional file 2: Figure S1).

#### PSA response

Two studies reported PSA response in mCRPC with HRD. Both olaparib monotherapy (RR: 5.85, 95% CI: 2.84–15.40) and cediranib plus olaparib (RR: 6.90, 95% CI: 1.86–28.36) showed significant improvement in PSA response (Additional file 2: Figure S2A). The combination therapy of olaparib plus cediranib had a higher SUCRA value (80.5%) compared to olaparib monotherapy (69.4%) (Additional file 2: Figure S2B). Sensitivity analysis showed consistent results (Additional file 2: Figure S3A).

#### Adverse events

Due to lack of data on adverse events for cediranib plus olaparib and olaparib plus AAP in the HRD cohort, we assessed the safety of different PARPis in both total cohort and HRD cohort. Olaparib showed a significantly lower all-grade AE rate compared with rucaparib (RR: 0.24, 95% CI: 0.01, 0.99) and cediranib plus olaparib (RR: 0.23, 95% CI: 0.01, 0.94) (Additional file 2: Figure S4A). Olaparib had the highest probability of being the regimen with the lowest all-grade AE rate (69.1%), followed by olaparib plus AAP (53.8%), rucaparib (14.5%) and cediranib plus olaparib (13%) in total cohort (Additional file 2: Figure S4B). In HRR-mutated cohort, two studies reported all-grade AEs. Olaparib showed a nonsignificant decrease in all-grade AEs compared with rucaparib (RR: 0.46, 95% CI: 0.06, 1.02) (Additional file 2: Figure S2C) and was ranked superior over rucaparib in HRD cohort (48.3% vs. 1.8%) (Additional file 2: Figure S2D). Sensitivity analysis showed consistent results (Additional file 2: Figure S3**B, S3C)**. Anemia and fatigue were the most commonly observed all-grades AEs in olaparib monotherapy, while for the combination therapy of olaparib plus AAP, nausea was most commonly reported. Fatigue was the most common all-grade AEs in rucaparib and cediranib plus olaparib.

#### Grade ≥ 3 AEs

In the total cohort, olaparib a showed significantly lower grade ≥ 3 AE rate compared with cediranib plus olaparib (RR: 0.72, 95% CI: 0.51, 0.97) (Additional file 2: Figure S4C) and had the highest probability to be the PARPi with lowest ≥ 3 grade AEs rate (63.3%), followed by rucaparib (54.1%), olaparib plus cediranib (17.9%) and olaparib plus AAP (15.3%) (Additional file 2: Figure S4D). In the HRR-mutated cohort, two studies reported grade ≥ 3 AEs. Olaparib showed a nonsignificant lower grade ≥ 3 AEs compared with rucaparib (RR: 0.95, 95% CI: 0.63, 1.39) (Additional file 2: Figure S2E) and was ranked superior over rucaparib (31.0% vs. 20.2%) (Additional file 2: Figure S2F). Sensitivity analysis showed consistent results (Additional file 2: Figure S3D, S3E). Anemia was the most commonly reported ≥ 3 grade AEs in olaparib, rucaparib and olaparib plus AAP, while hypertension was the most commonly observed ≥ 3 grade AEs in cediranib plus olaparib. The relative effect and SUCRA ranking of serious adverse events and dose adjustments in HRR-mutated population were also reported in the supplementary material (Additional file 3: Table S1, Additional file 4: Table S2). We have also drawn a comprehensive ranking plot integrating both efficacy and safety outcomes to help facilitate the selection of therapeutic options (Additional file 2: Figure S5).

#### Risk of bias evaluation

Three studies showed concerns in randomization process due to a lack of allocation concealment among participants, while two studies showed concerns regarding the deviation from intended interventions domain due to nonadherence of participants to their assigned treatment and lack of masking in patients and caregivers (Additional file 2: Figure S6).

## Discussion

This is the first network meta-analysis to indirectly compare the efficacy and safety of different PARPis on HRD mCRPC patients in the absence of head-to-head comparison in 2nd -line setting. This Bayesian NMA, based on four RCTs comprised of 1024 patients, illustrated the superiority of selected PARPi-based regimens for mCRPC with HRR mutation in the 2nd -line setting. However, there is no clear answer as to which therapy should be favored based on current available evidence. Although SUCRA values of rPFS and PSA response suggested that combination therapy of cediranib plus olaparib might be the most effective 2nd -line PARPi, the network meta-analysis based on Bayesian approach did not show this superiority to be statistically significant over any other PARPis. Moreover, significantly higher rates of all-grade and grade ≥ 3 AEs were found in this combination therapy compared with olaparib monotherapy, making safety a major concern.

Considering that PARPi mainly functioned by inducing synthetic lethality in patients with HRD, we did not include trials merely contained unselected population (e.g., PROpel, TALAPRO-2) [26, 27]. Next-generation hormone therapy (NHT) was discussed to (e.g., enzalutamide, abiraterone) potentially induce artificial DDR-insufficiency and caused a situation called “BRCA-ness”, explaining the prognostic benefit of concomitant usage of PARPi and ARAT in mCRPC patients compared to ARAT monotherapy [28]. It worth noting that not every patient can have an access to liquid biopsy or tissue-based genomic sequencing, so combination therapy of NHT and PARPi in unselected population may be considered. However, although both PROpel and TALAPRO-2 reported rPFS benefit in combination treatment cohort regardless of HRD status, we did not observe a significant increase in overall survival in PROpel (OR: 0·90, 95%CI: 0·72–1·13) in non-HRR-mutated cohort [26], while the final overall survival outcome for TALAPRO-2 in cohort without HRR mutation is unreported yet. Thus, the efficacy of PARPi or PARPi-based therapy in patients without HRR mutations should be interpreted with caution.

Subgroup analysis based on BRCA 1/2, BRCA-2 or ATM mutated population showed different ranking results, indicating that the selection of the optimal medication should be based on the genetic background of patients. We found PARPis had significantly superior efficacy over ARAT in patients with BRCA 1/2 mutations, while no significant survival difference was observed in ATM-mutated cohort. Our findings were similar to a recently published meta-analysis, which demonstrated the non-uniform efficacy of PARP inhibitors across mCRPC patients with DDR alterations [29]. Several previous studies have also shown limited efficacy of PARPis in mCRPC patients with ATM mutations [30] or in other cancer types (e.g., breast cancer, gastric and pancreatic cancer), but significant responses in cohort with BRCA 1/2 mutations [31–33]. As the most commonly mutated DDR genes, BRCA 1/2 serve as the key mediator in HRR pathway and can lead to HRD and induce tumor cell apoptosis and inhibition of tumorigenesis [34]. This explains our finding of the considerable response observed after PARPi treatments in BRCA 1/2-mutated cohort. However, ATM acts as a sensor for DDR rather than mediator of HR repair pathway [35, 36]. Therefore, ATM mutation alone might be insufficient to induce synthetic lethality, partially explaining the limited response in the ATM-mutated cohort. Moreover, the relatively low degree of biallelic loss, which is likely required for synthetic lethality, may also contribute to the low response rate of ATM-mutated patients to PARPi [37]. Although one previous study reported responses in ATM mutated mCRPC patients [38], the limited sample size restrained its credibility. Further large-scaled research is needed to synthesize more concrete evidence regarding the role ATM mutation plays in the effect of PARPi on mCRPC.

We found the combination therapy of olaparib and cediranib, a VEGFR TKI, had comparable therapeutic ranking compared to olaparib monotherapy in terms of PSA response and rPFS in HRD population. Angiogenesis is considered critical for the development of PCa [39], and PCa cells have been reported to exhibit high angiogenetic activity by producing VEGF, MMPs and TGF-β, et al [40, 41]. However, despite promising results observed in vitro and preclinical tumor models regarding the effect of angiogenesis inhibitors on PCa, none of the present phase III clinical trials regarding TKI monotherapy or combination therapies have met their clinical endpoints [42]. Besides, cediranib is currently not approved by Food and Drug Administration (FDA) for PCa and thus is not a standard treatment option for mCRPC. More large-scale randomized clinical trials are warranted regarding this field.

Due to the limitation of heterogeneity in terms of genetic mutational status in cohort of each trial, overall survival (OS) was not evaluated as a study outcome. None of the included studies reported significantly prolonged OS in intention-to-treat population. Factually, this was in accordance with the result of recently studies regarding PARPi-based combination therapy for mCRPC in both heavily pretreated biomarker-unselected population and the first-line setting with HRD [30, 43]. This might be explained by the crossover from control group to experimental group [9, 16, 17, 25]. Regarding this problem, Joseph W. et al. and M. Hussain et al. had conducted sensitivity analysis by excluding the cross-over. Neither of them showed significantly prolonged OS. However, M. Hussain et al. reported significantly improved OS in BRCA/ATM mutated subgroup before and after cross-over analysis [9, 16]. Considering that BRCA mutation constituted a proportion of over 50% in BRCA/ATM cohort in this study, and the significant role played by BRCA mutations in the synthetic lethality process, along with the suppressive effect on tumor cell proliferation brought by ATM mutation [44], the favorable therapeutic efficacy in OS achieved by olaparib over ARAT can be well explained. However, due to the difference existing in later-line treatment according to patients’ response and the immaturity of the survival data [25], OS achieved in the included studies should be further elucidated with caution.

We observed a significantly higher all-grade AE rate and grade ≥ 3 AE rate for cediranib plus olaparib compared with olaparib monotherapy in the total cohort analysis. Additionally, we noted a non-significant superiority of olaparib over olaparib plus AAP in safety regarding all-grade AEs and grade ≥ 3 AEs based on SUCRA values. As combination therapies were commonly accompanied with more adverse events during cancer treatment [45–47], caution should be exercised when determining the regimen selection. Although considerable efficacy can be achieved by combination therapies, higher concomitant incidence of AEs is directly associated with a lower quality of life of patients, dose adjustment and discontinuation. These factors may lead to shortened therapy course and decreased efficacy of combination therapies [16, 48].

Our findings should be interpreted with caution in context of the following limitations. Baseline characteristics of the included four RCTs were not completely consistent, and previous treatments varied. The efficacy and safety rankings of the combination therapy of cediranib plus olaparib and olaparib plus AAP were generate based on statistics from phase II trails with relatively small sample sizes. So, the ranking of these two combination therapies should be interpreted with caution. As eligible criterion in included studies varied in mutational status, we only chose cohort with HRD for data analysis. Not every study reported subgroup results, and the relative efficacy of the combination therapies may be dubious regarding the small HRD population reported [16]. The safety rankings of regimens in this study were generated based on HRD cohort or total cohort that did not specify the HRD status, the toxicity of PARPi in patients without HRR mutation should be further weighted. Some patients in one trial contained patients receiving more than one regimen prior to recruitment. Finally, most of the conclusions were based on indirect comparisons, so more head-to-head RCTs should be conducted in order to generate more concrete results.

## Conclusion

This Bayesian network meta-analysis provides the best current evidence available regarding the efficacy and safety of different PARPi-based therapies for mCRPC with HRR mutation in 2nd -line setting. PARPis showed considerable efficacy for HRD mCRPC. However, it is crucial to consider the genetic background of patients and potential adverse events when making treatment decisions. Head-to-head trials are warranted to further confirm these findings.

### Electronic supplementary material

Below is the link to the electronic supplementary material.

Supplementary Material 1

Supplementary Material 2

Supplementary Material 3

Supplementary Material 4

## Data Availability

The dataset analyzed for results of this study are available from the corresponding author upon reasonable request.

## Abbreviations

PCa: Prostate cancer
mCRPC: Metastatic castration-resistant prostate cancer
ARAT: Androgen receptor-axis-targeted
DDR: DNA damage repair
PARPi: Poly (ADP-ribose) polymerase inhibitors
HRD: Homologous recombination deficiency
HRR: Homologous recombination repair
NMA: Network meta-analysis
rPFS: Radiographic progression-free survival
PSA: Prostate specific antigen
AE: Adverse event
AEs: Adverse events
HR: Hazard ratio
RR: Risk ratio
DIC: Deviance information criterion
SUCRA: Surface under cumulative ranking
AAP: Acetate plus prednisone
