# Supplementary material for: The efficacy and safety of PARP inhibitors in mCRPC with HRR mutation in second-line treatment: a systematic review and bayesian network meta-analysis
Source: BMC Cancer. 2024 Jun 8;24:706. doi: 10.1186/s12885-024-12388-2 (PMC11162002; doi:10.1186/s12885-024-12388-2)
Supplement: Supplementary file 1 — Supplementary Material 1 [file 12885_2024_12388_MOESM1_ESM.pdf]

**Search strategy**

**PubMed/Medline**

1. Prostate cancer [Title/Abstract]
2. Prostate tumor [Title/Abstract]
3. Prostate carcinoma [Title/Abstract]
4. Prostate malignancy [Title/Abstract]
5. Prostate neoplasm [MeSH Terms]
6. Poly (ADP-ribose) polymerase [Title/Abstract]
7. PARP [Title/Abstract]
8. Olaparib [Title/Abstract]
9. Rucaparib [Title/Abstract]
10. Niraparib [Title/Abstract]
11. Talazoparib [Title/Abstract]
12. Veliparib [Title/Abstract]
13. Pamiparib [Title/Abstract]
14. 1 OR 2 OR 3 OR 4 OR 5
15. 6 OR 7 OR 8 OR 9 OR 10 OR 11 OR 12 OR 13
16. 14 AND 15

**EMBASE via Ovid interface**

1. Prostate cancer/
2. Prostate tumor/
3. Prostate malignancy.mp.
4. Prostate carcinoma/
5. Prostate neoplasm.mp.
6. PARP.mp.
7. Poly ADP ribose polymerase.mp
8. Olaparib/
9. Rucaparib/
10. Niraparib/
11. Talazoparib/
12. Veliparib/
13. Pamiparib/
14. 1 OR 2 OR 3 OR 4 OR 5
15. 6 OR 7 OR 8 OR 9 OR 10 OR 11 OR 12 OR 13
16. 14 AND 15

**Cochrane**

1. MeSH descriptor: [Prostatic Neoplasm] explode all trees
2. (Prostate cancer):ti,ab,kw
3. (Prostate tumor):ti,ab,kw
4. (Prostate malignancy):ti,ab,kw
5. (Prostate carcinoma):ti,ab,kw
6. MeSH descriptor: [Poly(ADP-ribose) Polymerase Inhibitors] explode all trees
7. (PARP inhibitor):ti,ab,kw
8. (Olaparib):ti,ab,kw
9. (Rucaparib):ti,ab,kw
10. (Niraparib):ti,ab,kw
11. (Talazoparib):ti,ab,kw
12. (Veliparib):ti,ab,kw
13. (Pamiparib):ti,ab,kw
14. #1 OR #2 OR #3 OR #4 OR #5
15. 15. #6 OR #7 OR #8 OR #9 OR #10 OR #11 OR #12 OR #13
16. #14 AND #15
