# Supplementary material for: The efficacy and safety of PARP inhibitors in mCRPC with HRR mutation in second-line treatment: a systematic review and bayesian network meta-analysis
Source: BMC Cancer. 2024 Jun 8;24:706. doi: 10.1186/s12885-024-12388-2 (PMC11162002; doi:10.1186/s12885-024-12388-2)
Supplement: Supplementary file 2 — Supplementary Material 2 [file 12885_2024_12388_MOESM2_ESM.docx]

***Additional File 1***

**Figure S1.** Sensitivity analysis of radiographic progression-free survival based on mutational status in terms of SUCRA. A: Sensitivity analysis of radiographic progression-free survival for HRR-mutated population in terms of SUCRA; B: Sensitivity analysis of radiographic progression-free survival in BRCA 1/2-mutated population; C: Sensitivity analysis of radiographic progression-free survival in BRCA2-mutated population; D: Sensitivity analysis of radiographic progression-free survival in ATM-mutated population. Abbreviation: SUCRA: surface under cumulative ranking; HRR: homologous recombination repair; ARAT: androgen receptor-axis-targeted therapy; CED: cediranib; OLA: olaparib; AAP: abiraterone acetate plus prednisone; RUCA: rucaparib.

**Figure S2.** The pairwise comparison and SUCRA for secondary outcomes in HRR-mutated population. A: The league table for PSA response in HRR-mutated population; B: SUCRA for PSA response in HRR-mutated population; C: The league table for all-grade AEs in HRR-mutated population; D: SUCRA for all-grade AEs in HRR-mutated population; E: The league table for grade ≥ 3 AEs in HRR-mutated population; F: SUCRA for grade≥3 AEs HRR-mutated population. Abbreviation: SUCRA: surface under cumulative ranking; PSA: prostate specific antigen; AEs: adverse events; HRR: homologous recombination repair; ARAT: androgen receptor-axis-targeted therapy; CED: cediranib; OLA: olaparib; RUCA: rucaparib.

**Figure S3.** Sensitivity analysis of secondary outcomes in terms of SUCRA. A: Sensitivity analysis of PSA response in HRR-mutated population; B: Sensitivity analysis of all-grade AEs in HRR-mutated population; C: Sensitivity analysis of all-grade AEs in total cohort; D: Sensitivity analysis of grade≥3 AEs in HRR-mutated population; E: Sensitivity analysis of grade≥3 AEs in total cohort. Abbreviation: SUCRA: surface under cumulative ranking; AEs: adverse events; HRR: homologous recombination repair; ARAT: androgen receptor-axis-targeted therapy; CED: cediranib; OLA: olaparib; AAP: abiraterone acetate plus prednisone; RUCA: rucaparib.

**Figure S4.** The pairwise comparison and SUCRA for AEs in total cohort. A: The league table for all-grade AEs in total cohort; B: SUCRA for all-grade AEs in total cohort; C: The league table for grade≥3 AEs in total cohort; D: SUCRA for grade≥3 AEs in total cohort. Abbreviation: SUCRA: surface under cumulative ranking; AEs: adverse events; ARAT: androgen receptor-axis-targeted therapy; CED: cediranib; OLA: olaparib; AAP: abiraterone acetate plus prednisone; RUCA: rucaparib.

**Figure S5.** Rankings of the regimens reported in the included studies based on rPFS (A), all-grade AEs (B), ≥3 grade AEs (C) in total cohort and the all-grade and ≥3 grade AEs in HRRm subgroup cohort (D, E). Abbreviations: rPFS: radiographic progression-free survival; AEs: adverse events; HRRm: homologous repair recombination mutated; OLA: olaparib; RUCA: rucaparib; CEDplusOLA: cediranib plus olaparib; OLAplusAAP: olaparib plus abiraterone acetate plus prednisone.

**Figure S6.** Risk of bias assessment of all included studies. Abbreviation: ARAT: androgen receptor-axis-targeted therapy; AAP: abiraterone acetate plus prednisone.

**Figure S1.**


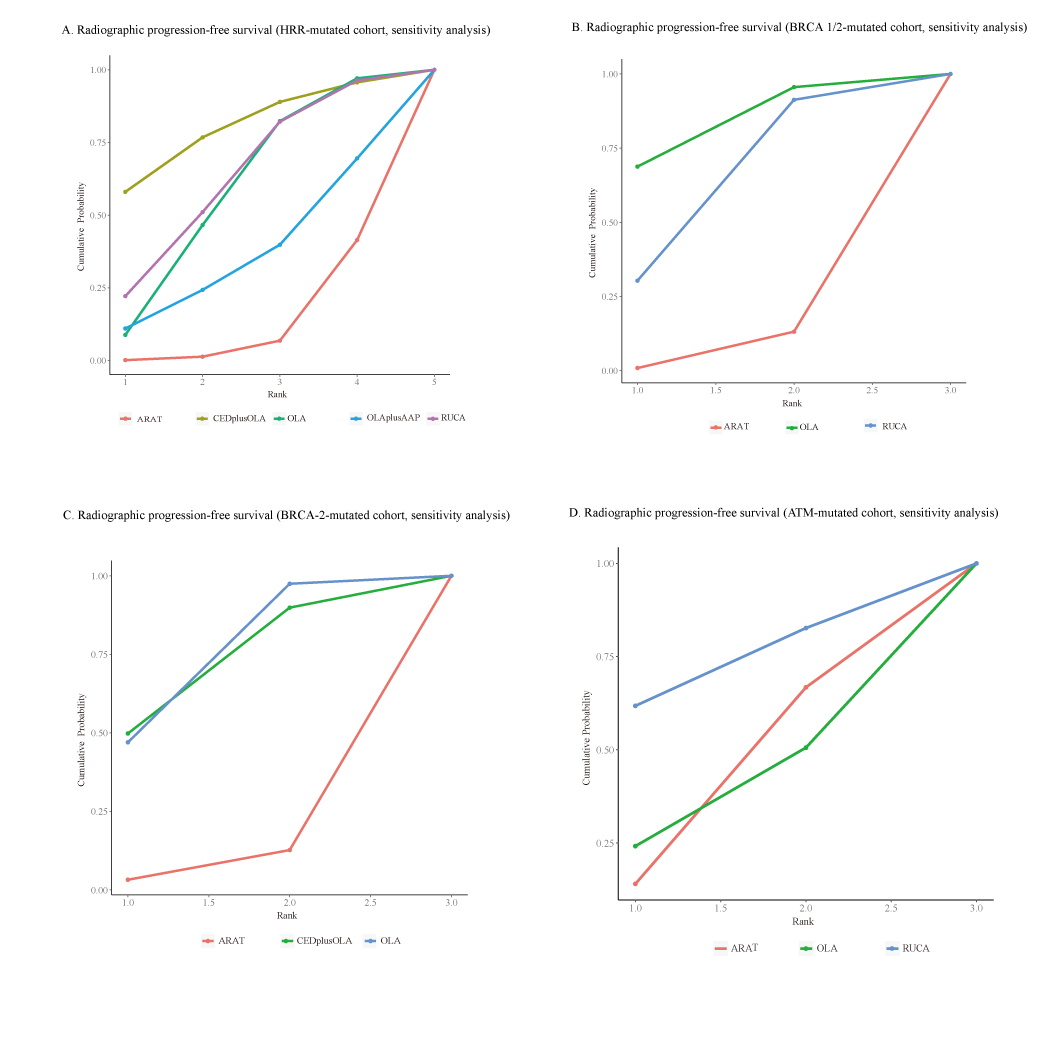


Sensitivity analysis of radiographic progression-free survival based on mutational status in terms of SUCRA. A: Sensitivity analysis of radiographic progression-free survival for HRR-mutated population in terms of SUCRA; B: Sensitivity analysis of radiographic progression-free survival in BRCA 1/2-mutated population; C: Sensitivity analysis of radiographic progression-free survival in BRCA2-mutated population; D: Sensitivity analysis of radiographic progression-free survival in ATM-mutated population. Abbreviation: SUCRA: surface under cumulative ranking; HRR: homologous recombination repair; ARAT: androgen receptor-axis-targeted therapy; CED: cediranib; OLA: olaparib; AAP: abiraterone acetate plus prednisone; RUCA: rucaparib.

**Figure S2.**

**
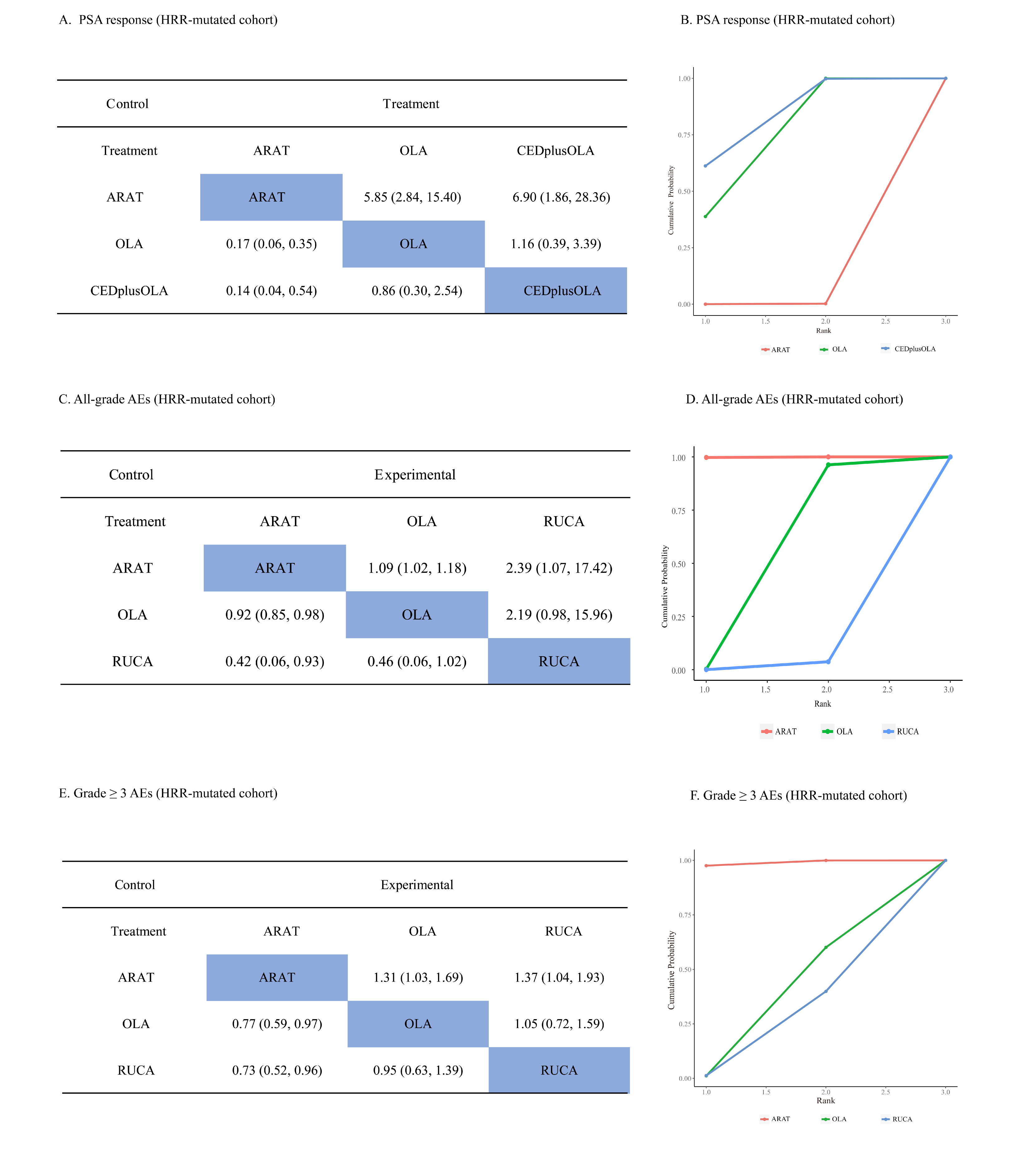
**

The pairwise comparison and SUCRA for secondary outcomes in HRR-mutated population. A: The league table for PSA response in HRR-mutated population; B: SUCRA for PSA response in HRR-mutated population; C: The league table for all-grade AEs in HRR-mutated population; D: SUCRA for all-grade AEs in HRR-mutated population; E: The league table for grade ≥ 3 AEs in HRR-mutated population; F: SUCRA for grade≥3 AEs HRR-mutated population. Abbreviation: SUCRA: surface under cumulative ranking; PSA: prostate specific antigen; AEs: adverse events; HRR: homologous recombination repair; ARAT: androgen receptor-axis-targeted therapy; CED: cediranib; OLA: olaparib; RUCA: rucaparib.

**Figure S3.**

**
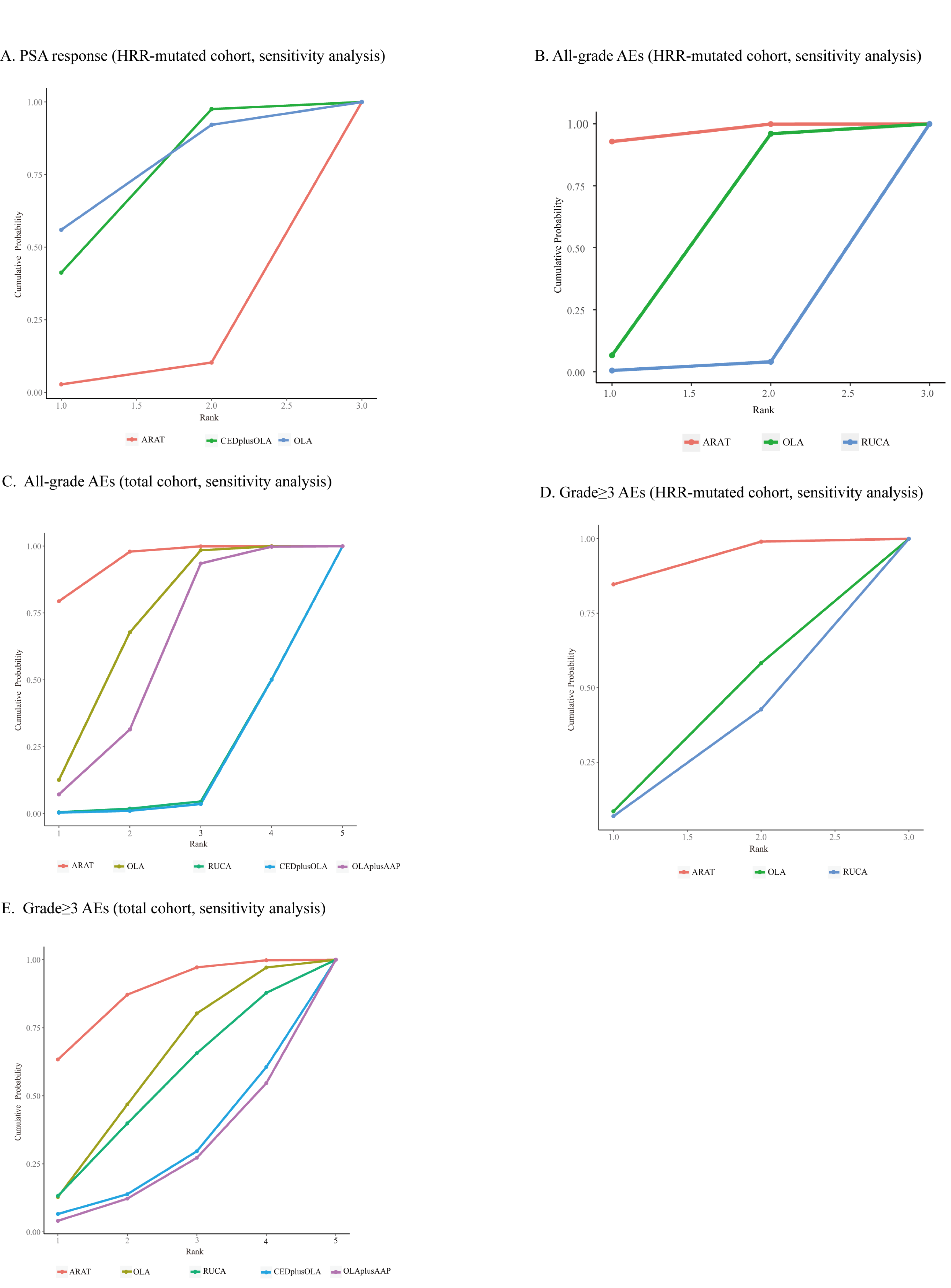
**

Sensitivity analysis of secondary outcomes in terms of SUCRA. A: Sensitivity analysis of PSA response in HRR-mutated population; B: Sensitivity analysis of all-grade AEs in HRR-mutated population; C: Sensitivity analysis of all-grade AEs in total cohort; D: Sensitivity analysis of grade≥3 AEs in HRR-mutated population; E: Sensitivity analysis of grade≥3 AEs in total cohort. Abbreviation: SUCRA: surface under cumulative ranking; AEs: adverse events; HRR: homologous recombination repair; ARAT: androgen receptor-axis-targeted therapy; CED: cediranib; OLA: olaparib; AAP: abiraterone acetate plus prednisone; RUCA: rucaparib.

**Figure S4.**

**
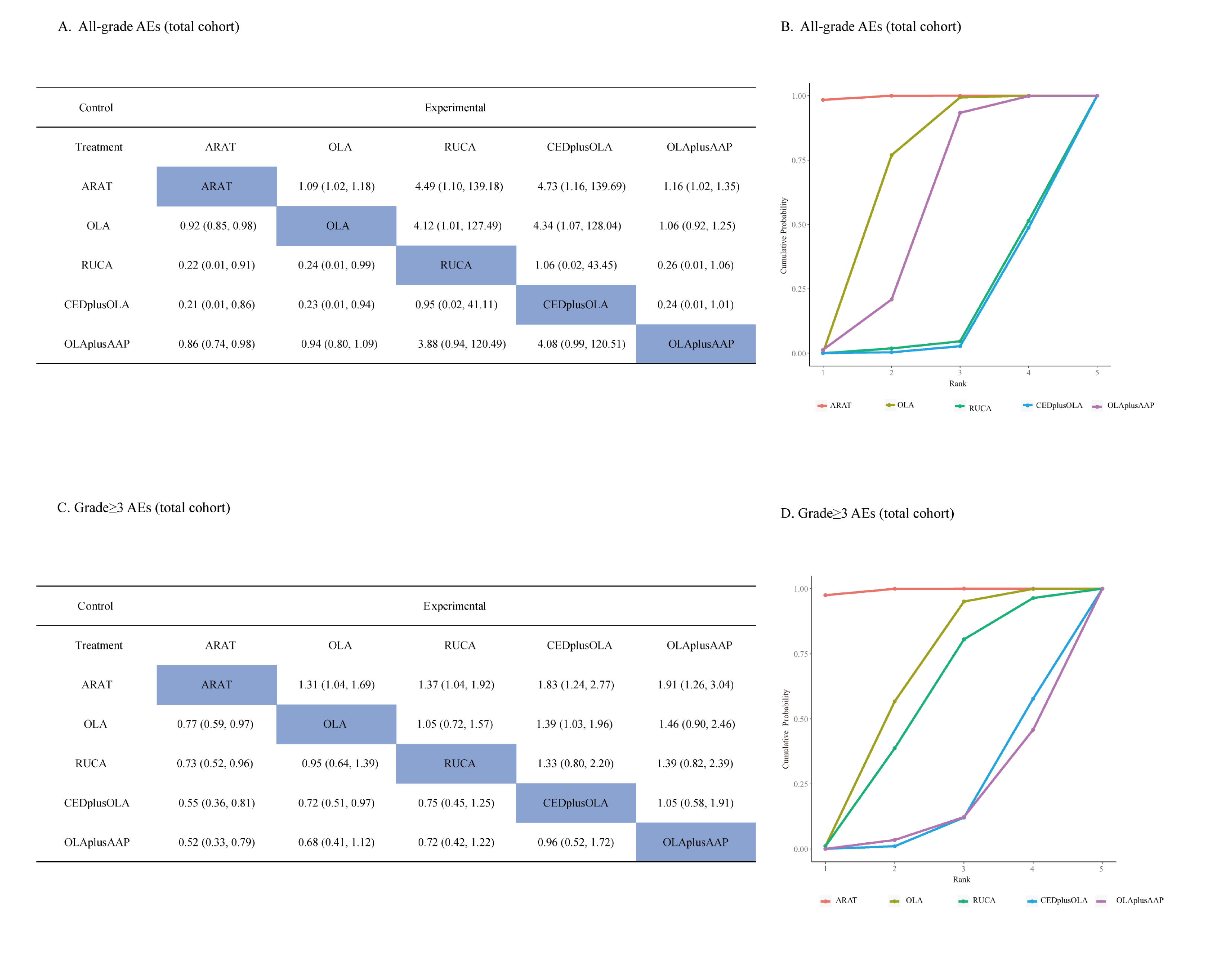
**

The pairwise comparison and SUCRA for AEs in total cohort. A: The league table for all-grade AEs in total cohort; B: SUCRA for all-grade AEs in total cohort; C: The league table for grade≥3 AEs in total cohort; D: SUCRA for grade≥3 AEs in total cohort. Abbreviation: SUCRA: surface under cumulative ranking; AEs: adverse events; ARAT: androgen receptor-axis-targeted therapy; CED: cediranib; OLA: olaparib; AAP: abiraterone acetate plus prednisone; RUCA: rucaparib.

**Figure S5.**


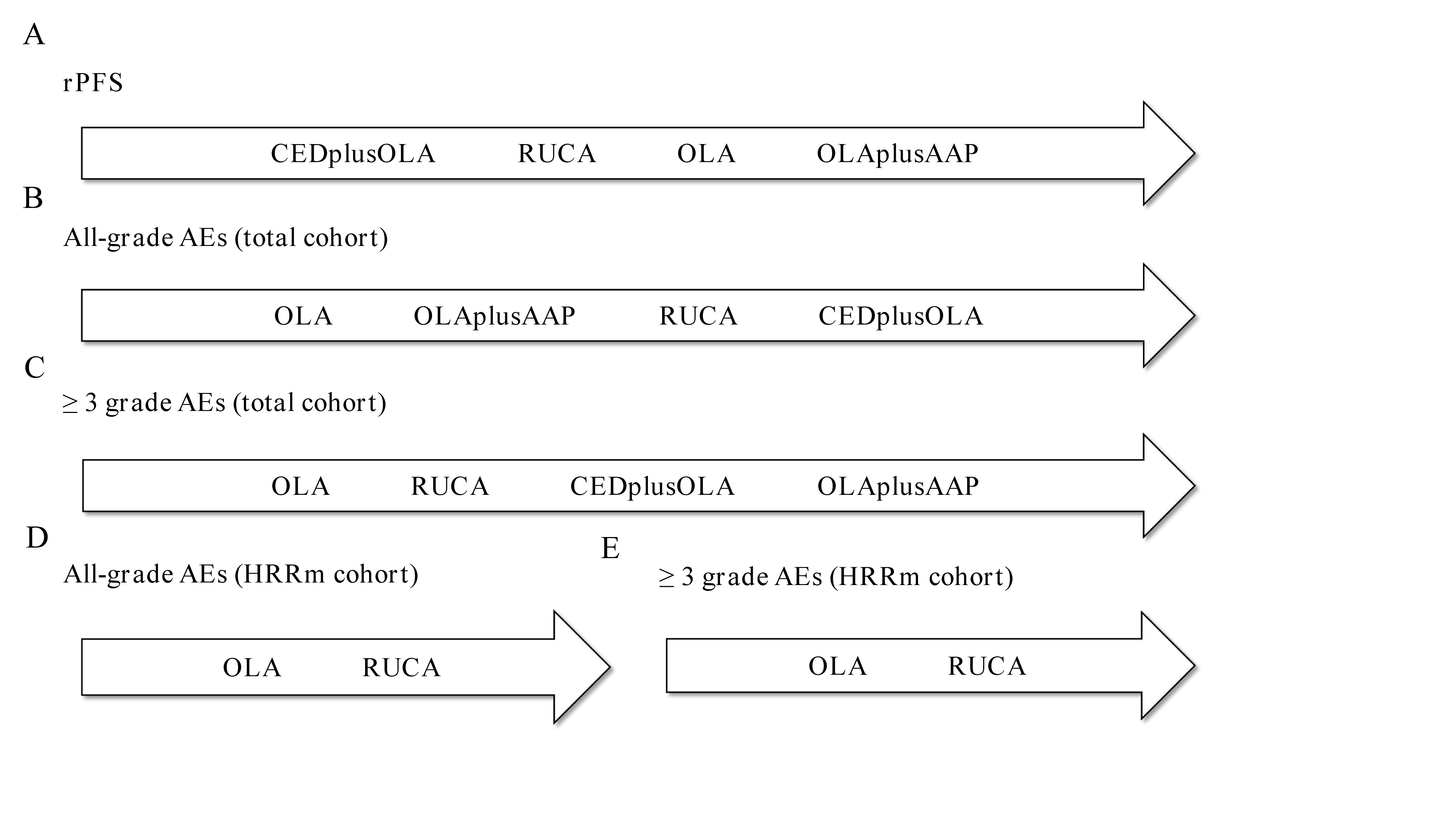


Rankings of the regimens reported in the included studies based on rPFS (A), all-grade AEs (B), ≥3 grade AEs (C) in total cohort and the all-grade and ≥3 grade AEs in HRRm subgroup cohort (D, E). Abbreviations: rPFS: radiographic progression-free survival; AEs: adverse events; HRRm: homologous repair recombination mutated; OLA: olaparib; RUCA: rucaparib; CEDplusOLA: cediranib plus olaparib; OLAplusAAP: olaparib plus abiraterone acetate plus prednisone.

**Figure S6.**

**
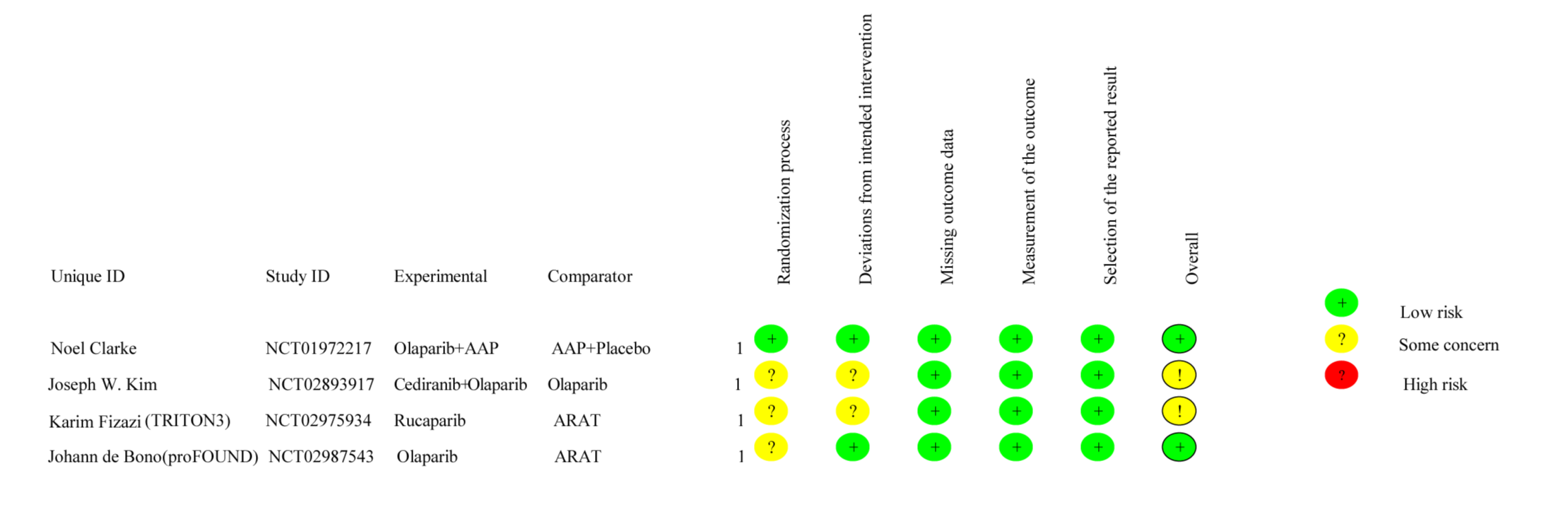
**

Risk of bias assessment of all included studies. Abbreviation: ARAT: androgen receptor-axis-targeted therapy; AAP: abiraterone acetate plus prednisone.
