# Supplementary material for: The efficacy and safety of PARP inhibitors in mCRPC with HRR mutation in second-line treatment: a systematic review and bayesian network meta-analysis
Source: BMC Cancer. 2024 Jun 8;24:706. doi: 10.1186/s12885-024-12388-2 (PMC11162002; doi:10.1186/s12885-024-12388-2)
Supplement: Supplementary file 3 — Supplementary Material 3 [file 12885_2024_12388_MOESM3_ESM.docx]

**Table S1.**

| RR (compared with ARAT) | Treatment | Olaparib | Rucaparib |
| --- | --- | --- | --- |
| Serious adverse event or dose adjustment | SAE | 1.2 (0.91, 1.7) | 1.3 (0.83, 2.4) |
|  | Interruption of intervention owing to adverse event | 2.4 (1.7, 3.7) | 2.7 (1.7, 4.9) |
|  | Dose reduction owing to adverse event | 4.5 (2.3, 11) | 0.82 (0.46, 1.6) |
|  | Discontinuation owing to adverse event | 2.4 (1.3, 4.7) | 1.9 (0.83, 5.3) |
|  | Death from adverse event | 0.86 (0.32, 2.6) | 0.38 (0.093, 1.9) |

Relative effect for serious adverse events or dose adjustment in HRR-mutated population based on network meta-analysis. Abbreviation: ARAT: androgen receptor-axis-targeted therapy; RR: risk ratio; SAE: serious adverse event.
