# Supplementary material for: The efficacy and safety of PARP inhibitors in mCRPC with HRR mutation in second-line treatment: a systematic review and bayesian network meta-analysis
Source: BMC Cancer. 2024 Jun 8;24:706. doi: 10.1186/s12885-024-12388-2 (PMC11162002; doi:10.1186/s12885-024-12388-2)
Supplement: Supplementary file 4 — Supplementary Material 4 [file 12885_2024_12388_MOESM4_ESM.docx]

**Table S2.**

| SUCRA score | Fixed-effects model | | | | | Random-effects model (sensitivity analysis) | | | |
| --- | --- | --- | --- | --- | --- | --- | --- | --- | --- |
|  | Treatment | Olaparib | Rucaparib | ARAT | Ranking | Olaparib | Rucaparib | ARAT | Ranking |
| Serious adverse event or dose adjustment | SAE | 0.345 | 0.263 | 0.892 | ARAT>olaparib>rucaparib | 0.376 | 0.277 | 0.847 | ARAT>olaparib>rucaparib |
|  | Interruption of intervention owing to adverse event | 0.302 | 0.198 | 1.000 | ARAT>olaparib>rucaparib | 0.308 | 0.244 | 0.948 | ARAT>olaparib>rucaparib |
|  | Dose reduction owing to adverse event | 0.000 | 0.867 | 0.633 | Rucaparib>ARAT>olaparib | 0.062 | 0.772 | 0.667 | Rucaparib>ARAT>olaparib |
|  | Discontinuation owing to adverse event | 0.161 | 0.375 | 0.964 | ARAT>rucaparib>olaparib | 0.217 | 0.383 | 0.900 | ARAT>rucaparib>olaparib |
|  | Death from adverse event | 0.400 | 0.849 | 0.251 | Rucaparib>olaparib>ARAT | 0.411 | 0.799 | 0.290 | Rucaparib>olaparib>ARAT |

SUCRA for serious adverse events and dose adjustment in HRR-mutated population and the corresponding sensitivity analysis. Abbreviation: ARAT: androgen receptor-axis-targeted therapy; SUCRA: surface under the cumulative ranking; SAE: serious adverse event.
